# Transcription Factors Mat2 and Znf2 Operate Cellular Circuits Orchestrating Opposite- and Same-Sex Mating in *Cryptococcus neoformans*

**DOI:** 10.1371/journal.pgen.1000953

**Published:** 2010-05-13

**Authors:** Xiaorong Lin, Jennifer C. Jackson, Marianna Feretzaki, Chaoyang Xue, Joseph Heitman

**Affiliations:** 1 Department of Biology, Texas A&M University, College Station, Texas, United States of America; 2 Department of Molecular Genetics and Microbiology, Duke University Medical Center, Durham, North Carolina, United States of America; 3 Public Health Research Institute, New Jersey Medical School, University of Medicine and Dentistry of New Jersey, Newark, New Jersey, United States of America; Washington University School of Medicine, United States of America

## Abstract

*Cryptococcus neoformans* is a human fungal pathogen that undergoes a dimorphic transition from a unicellular yeast to multicellular hyphae during opposite sex (mating) and unisexual reproduction (same-sex mating). Opposite- and same-sex mating are induced by similar environmental conditions and involve many shared components, including the conserved pheromone sensing Cpk1 MAPK signal transduction cascade that governs the dimorphic switch in *C. neoformans*. However, the homeodomain cell identity proteins Sxi1α/Sxi2**a** encoded by the mating type locus that are essential for completion of sexual reproduction following cell–cell fusion during opposite-sex mating are dispensable for same-sex mating. Therefore, identification of downstream targets of the Cpk1 MAPK pathway holds the key to understanding molecular mechanisms governing the two distinct developmental fates. Thus far, homology-based approaches failed to identify downstream transcription factors which may therefore be species-specific. Here, we applied insertional mutagenesis via *Agrobacterium*-mediated transformation and transcription analysis using whole genome microarrays to identify factors involved in *C. neoformans* differentiation. Two transcription factors, Mat2 and Znf2, were identified as key regulators of hyphal growth during same- and opposite-sex mating. Mat2 is an HMG domain factor, and Znf2 is a zinc finger protein; neither is encoded by the mating type locus. Genetic, phenotypic, and transcriptional analyses of Mat2 and Znf2 provide evidence that Mat2 is a downstream transcription factor of the Cpk1 MAPK pathway whereas Znf2 functions as a more terminal hyphal morphogenesis determinant. Although the components of the MAPK pathway including Mat2 are not required for virulence in animal models, Znf2, as a hyphal morphology determinant, is a negative regulator of virulence. Further characterization of these elements and their target circuits will reveal genes controlling biological processes central to fungal development and virulence.

## Author Summary

Although sexual reproduction typically involves partners of opposite mating type (sexuality), sex can occur with just one mating type and even with single individuals (parthenogenesis, homothallism). For example, *Cryptococcus neoformans*, a fungal pathogen that causes cryptococcal meningitis, can undergo opposite-sex mating and same-sex mating. The ability to undergo bisexual and unisexual mating provides this fungus a unique opportunity to maintain its ability to undergo sexual reproduction in largely unisexual natural populations (α). However, the molecular mechanisms underlying these two sexual reproduction processes are unclear. By random mutagenesis and gene expression profiling, we have identified two key transcription factors, Mat2 and Znf2, that operate cellular circuits orchestrating opposite- and same-sex mating in *C. neoformans*. The findings presented here provide a foundation to further elucidate the circuits evoking two different modes of sexual reproduction and to investigate the relationship between morphological differentiation and virulence in this ubiquitous pathogen. Recent studies suggest that unisexual mating might occur in several major human pathogenic fungi, and thus knowledge about the molecular mechanisms controlling the two sexual reproduction modes in *C. neoformans* may also provide insights on the evolution of bifurcate mating systems in other organisms.

## Introduction

Many fungi undergo dramatic morphological differentiation during their life cycles. The morphological transition between the yeast form and the pseudohyphal form during mating and invasive growth in *Saccharomyces cerevisiae* has served as a paradigm for developmental biology due to the well-characterized genetics and robust molecular tools in this organism. For example, the mitogen-activated protein kinase (MAPK) cascade regulating the dimorphic switch in *S. cerevisiae*, often referred to as the pheromone response pathway, provides a framework for studying morphogenesis in a variety of fungal species, including the human fungal pathogen *Cryptococcus neoformans* [1]–[5]. Homologs of the core components of the MAPK cascade are conserved among evolutionarily distantly related fungi. However, the downstream targets of the MAPK cascade, which are effectors that ultimately evoke species-specific adaptive responses to external or internal signals, are often not conserved. Therefore, the identity of the downstream transcription factors that activate or repress corresponding target genes in different species are often difficult to reveal through a candidate gene approach.

Dimorphism is a prominent feature shared by the majority of pathogenic fungi that can cause systemic mycoses in human and animals, such as *Blastomyces dermatitidis*, *Candida albicans*, *Coccidioides immitis*, *Histoplasma capsulatum*, *Paracoccidioides brasiliensis*, *Penicillium marneffei*, and *Sporothrix schenkii*. The ability to switch between the unicellular yeast form and the multicellular hyphal form has been actively investigated in these fungi because dimorphism is not only an important aspect of fungal development but also integral to fungal pathogenicity [6]–[19].

Unlike the majority of other human fungal pathogens, *C. neoformans* has been typically considered as a yeast and not a dimorphic fungus. In addition, *C. neoformans* belongs to the Basidiomycota and is more closely related to mushrooms in an evolutionary sense than to the dimorphic fungal pathogens mentioned above that belong to the phylum of Ascomycota. This fungus can cause fatal cryptococcal meningitis in predominantly immunocompromised hosts and also, less frequently, in immunocompetent individuals [20]–[23]. It is second only to tuberculosis in mortality burden in AIDS patients worldwide [24].

*C. neoformans* yeast cells differentiate into a hyphal form during opposite sex mating and same sex mating. This heterothallic fungus has two opposite mating types: **a** or α, and opposite sex mating initiates when haploid **a** and α yeast cells undergo cell-cell fusion [25]–[28]. The two parental nuclei remain separated after the cell-cell fusion event and the resulting **a**-α dikaryon initiates a morphological switch to dikaryotic hyphal growth with clamp cells connecting neighboring hyphal compartments, which ensures the inheritance of both parental nuclei in each hyphal cell [29], [30]. Nuclear fusion followed by meiosis occurs in swollen aerial hyphal tips (basidia). Four chains of basidiospores are subsequently generated [27], [28]. This **a**-α mating initiated hyphal growth and basidiospore production has been observed in both serotypes A and D of *C. neoformans* and also in the sibling species *C. gattii* [25], [26], [31], [32].

Hyphal growth can also occur through same sex mating (also called monokaryotic fruiting) under conditions similar to those that induce **a**-α mating. Same sex mating involves cells of only one mating type, commonly α, and has been observed under laboratory conditions, mostly in the serotype D lineage [30], [33]–[35] and rarely in serotype A [36]. Hyphae generated during this process contain one nucleus per hyphal compartment with unfused clamp cells [30], [33]–[35], [37]. During same sex mating, nuclear diploidization could be accomplished by either cell-cell fusion between cells of the same mating type (e.g. α-α mating) or endoreplication [30], [38], and this ploidy increase could occur at multiple developmental stages prior to meiosis in the basidia. Reduction to the haploid state through meiosis in the basidia and generation of haploid spores during unisexual reproduction is similar to processes occurring during traditional **a**-α mating [30].

The homeodomain cell identity protein complex Sxi1α/Sxi2**a** encoded by the mating type locus is essential for **a**-α mating. This protein complex initiates dikaryotic specific events and regulates **a**-α mating after the cell fusion event, but neither Sxi1α nor Sxi2**a** is required for same sex mating [39], [40] (Lin X and Heitman J, unpublished results). Thus, same sex mating occurs without the Sxi1α or the Sxi2**a** protein. In *S. cerevisiae* and the related pathogenic yeast *Candida albicans*, the **a**/α cell type and **a**-α mating is controlled by the **a**1/α2 homeodomain heterodimeric complex. Remarkably, *Candida lusitaniae* and *Candida guilliermondii*, two haploid species with extant complete sexual cycles, are able to undergo sexual reproduction, yet both species lack the α2 gene and *C. guilliermondii* is also missing the **a**1 gene. Recent studies on the evolution of mating type determination and sexual reproduction of pathogenic *Candida* species have revealed considerable plasticity in the configuration of the mating type locus and the related cellular circuits that govern the establishment of cell type identity and promote sexual reproduction [41]–[44]. By analogy, same sex mating in *C. neoformans* could involve unique cellular circuits that evoke same sex mating and thereby bypass the central regulatory role of the Sxi1α/Sxi2**a** complex in sexual reproduction.

The morphological transition from the yeast to the hyphal form during both opposite and same sex mating is governed by the Cpk1 MAPK (mitogen-activated protein kinase) pheromone response signaling pathway [4]. This MAPK pathway controlling development is structurally and functionally conserved among different fungi, including *S. cerevisiae* [1]–[5], [45]. This pathway involves sequential activation of PAK (p21-activated kinase Ste20), MEKK (MAPK kinase kinase Ste11), MEK (MAPK kinase Ste7), and MAPK (Cpk1) [4]. The *C. neoformans* homologs of this cascade have been identified and shown to effect the dimorphic transition during mating [4], [46]–[48].

In *S. cerevisiae*, the downstream transcription factor of the pheromone response MAPK cascade is the homeodomain protein Ste12 [49], [50]. *STE12* homologs have also been identified in *C. neoformans* and are encoded by the mating type locus [51]–[54]. Although overexpression of *STE12*α induces pheromone production and deletion of the gene results in defective monokaryotic fruiting [51]–[53], disruption of *STE12* does not abolish pheromone sensing or opposite sex mating in contrast to other components of the MAPK cascade [4], [52], [53](Figure S1). Thus in *C. neoformans* Ste12 does not appear to be the sole or major target of the Cpk1 pathway, and instead likely functions in a branched or parallel signaling pathway [4]. In the ascomyceteous dimorphic fungus *Penicillium marneffei*, the Ste12 homolog stlA is also dispensable for dimorphic switching [55]. Studies of mating pathways in other fungal species also indicate that the key downstream transcription factors are often not conserved across different fungal lineages. Interestingly, HMG domain proteins are frequently the downstream transcription factors in pheromone sensing and mating cascades. Examples include Ste11 in the ascomyceteous yeast *Schizosaccharomyces pombe* [56] and Prf1 in the basidiomycetous dimorphic plant pathogen *Ustilago maydis* [57], [58]. *C. neoformans* and *U. maydis* are evolutionarily related, yet deletion of the *PRF1* homolog in several *C. neoformans* strain backgrounds does not affect opposite or same sex mating (Lin X, Kraus P, Hicks J, and Heitman J, unpublished results), indicating this HMG protein is not the MAPK target in *C. neoformans*, further supporting the species-specific nature of effector transcription factors.

Genes encoded by the mating type locus (>20) of *C. neoformans* play central roles in dimorphic hyphal growth. For example, the mating type locus encodes several key mating elements, including the pheromones (MFα, MF**a**), pheromone receptors (Ste3α and Ste3**a**), and components of the Cpk1 MAPK pathway such as Ste11 and Ste20 [30], [40], [47], [48], [59]–[62]. Whereas the Cpk1 MAPK pathway controls both **a**-α and α-α mating, the Sxi1α/Sxi2**a** complex is only specifically required for **a**-α mating. No distinct molecules specifically involved in α-α mating have been discovered and whether the Cpk1 MAPK pathway regulates **a**-α mating through the Sxi1α/Sxi2**a** complex also remained to be tested. Moreover, no apparent candidate downstream effector of the Cpk1 MAPK pathway appears to be encoded by the mating type locus.

Dimorphism and pathogenicity are intimately related in many dimorphic fungi and genes involved in dimorphism often regulate virulence [6]–[19]. For example, in *C. albicans*, a common human pathogen related to *S. cerevisiae*, the Cph1 (Ste12 homolog) MAPK pathway contributes to virulence [18], [63]–[65]. Similarly, the Prf1 MAPK pathway regulates both dimorphic growth and pathogenicity in the plant pathogen *U. maydis* [57], [66], [67]. Whether such a relationship also exists in *C. neoformans* needs to be tested.

The objectives of this study are to identify transcription factors downstream of the pheromone sensing Cpk1 MAPK pathway and to examine (1) if they play distinct roles in **a**-α and α-α mating and (2) if they are required for *Cryptococcus* virulence. Here, novel transcription factors involved in dimorphic hyphal growth of *C. neoformans* were identified via genetic and genomic approaches. First, genes highly expressed in a hyperfilamentous strain were identified by microarray analysis. Second, genes required for filamentation were identified by isolating mutants locked in the yeast phase following insertional mutagenesis. These approaches led to the identification of two transcription factors: Mat2 and Znf2, which are key regulators of **a**-α and α-α mating. Mat2 is an effector transcription factor of the Cpk1 MAPK pathway, whereas Znf2 functions as a more terminal hyphal morphology determinant. Like other components of the Cpk1 pathway, Mat2 is dispensable for virulence. Interestingly, deletion of Znf2 locks cells in the yeast phase and also enhances virulence. Our results suggest that although components in the signal transduction pathway may not regulate virulence, the inherent ability to grow in different morphotypes does affect *Cryptococcus* pathogenicity. Together, these findings provide a foundation to elucidate the circuits evoking two different modes of sexual reproduction in *C. neoformans* and to investigate the relationship between dimorphism and virulence in this ubiquitous human pathogen.

## Results

### Identification of the HMG protein Mat2 orchestrating hyphal growth via insertional mutagenesis

Because the transcription factors controlled by pheromone sensing MAPK pathways are species-specific and difficult to identify, we employed insertional mutagenesis via *Agrobacterium*-mediated transformation (AMT), an approach successfully applied in *C. neoformans* to identify genes regulating virulence traits [68]–[70]. Here this approach was applied to identify genes required for dimorphic hyphal growth. As the Cpk1 MAPK pathway regulates both **a**-α mating and α-α unisexual mating processes, and disruption of its components severely compromises dimorphic hyphal growth, we hypothesized that the transcription factor target of this pathway would be critical for filamentation during both processes. Because only one cell type is involved in unisexual reproduction, we screened for mutants defective in the transition from yeast to hyphae during α-α unisexual reproduction.

A total of 3600 insertional mutants were generated in a hyperfilamentous strain (XL280α) background. XL280α is an F1 progeny from a cross between strains B3501α and B3502**a** that share ∼75% genetic contents with each other [35]. The complete genome sequences of both B3501 and B3502 (congenic with JEC21 and isogenic with JEC20) are known [71]. Strain XL280α produces abundant hyphae on a variety of filamentation inducing media (SLAD, Filament Agar, and V8 agar) (data not shown) [35]. The insertional mutants generated were incubated on V8 agar medium at room temperature in the dark and screened for filamentation defects by microscopic examination. Six mutants that consistently displayed no hyphal growth on filamentation inducing media were selected. The insertion sites of these mutants were identified using inverse PCR and sequencing as described previously [68]. Sequences obtained were used in BLAST searches of the *C. neoformans* genome databases for the serotype D reference strain JEC21 to identify the insertion site and consequently, the disrupted genes (Table 1). The MAPK kinase Ste7 that signals directly upstream of the MAP kinase Cpk1 was identified via this approach (Table 1).

**Table 1:** Genes necessary for filamentation based on insertional mutant phenotype.

Locus name	Size (aa)	Chr	Protein Name	Identifiable motifs	Close homolog	Subcellular localization
164.m01417 (*MAT2*)	661	13	Hypothetical protein	One HMG domain at N-terminus	MAT-2 protein of *Fusarium oxysporum*	Nuclear, Bipartite NLS
177.m03335	198	7	Putative vacuole fusion, non-autophagic-related protein	3 transmembrane domains	cdc42 negative regulator nrf1in *S. pombe*	ER membrane retention
179.m00215	510	3	MAP kinase kinase	Ste7	Ste7 in *C. neoformans*	cytoplasmic
179.m00343	219	3	Expressed protein	N/A	cytokine inducing-glycoprotein in *C. neoformans*	extracellular
181.m08056	221	1	Conserved protein	N/A	Hypothetical protein SPAC869.06c in *S. pombe*	mitochondrial
186.m03696	858	2	Golgi to plasma membrane transport-related protein	One EF hand calcium binding domain	Retinitis pigmentosa GTPase regulator-like protein in *Takifugu rubripes*	nuclear

Information about size and chromosome location (Chr) were obtained from the JEC21 genome database hosted at TIGR [71]. Motif prediction was based on information of the JEC21 genome database and the motif scan tool as described previously (http://myhits.isb-sib.ch/cgi-bin/motif_scan) [114]. Subcellular localization was predicted using the WoLF PSORT program as described previously (http://wolfpsort.org/) [115].

Of the six genes identified, only 164.m01417 is likely to encode a transcription factor due to the presence of an HMG domain. The N-terminal HMG domain of the predicted protein shares similarity with the *Fusarium oxysporum* MAT-2 protein and was therefore named *Cryptococcus MAT2*. The *MAT2* gene is located on chromosome 13 and is unlinked to the mating type locus located on chromosome 4. Because HMG domain proteins have been found to be central transcription factors governning mating in many fungi [72], and because *MAT-2* genes are well-known regulators of mating encoded by the mating type locus in many other fungal species [73]–[75], the *Cryptococcus MAT2* gene was chosen for further characterization in this study.

### A zinc finger protein, Znf2, is highly expressed during hyphal growth

We hypothesized that, like the components of the Cpk1 MAPK cascade, the downstream transcription factors of this pathway function as positive regulators of **a**-α and α-α mating, and are upregulated during transitions to hyphal growth. Therefore, genes highly expressed in the hyperfilamentous strain XL280α compared to a non-filamentous strain XL34α under filamentation inducing conditions were identified by microarray profiling [76]. Like strain XL280α, strain XL34α is also an F1 progeny from the cross between strains B3510α and B3502**a** [35]. However, XL34α remains in the yeast form even under hyphal inducing conditions and is non-filamentous (data not shown). The comparison of the transcriptome profiles of XL280α and XL34α revealed 24 genes that exhibited three-fold or higher expression levels in XL280α compared to XL34α (Table 2). Not surprisingly, eight genes are encoded by the α mating type locus. The *CPK1* gene encoding the pheromone sensing MAP kinase unlinked to the mating type locus was also highly expressed. Three genes encoding potential transcription factors were identified: *ZNF1*α located in the mating type locus, *ZNF2* resident in another genomic region, and the well-characterized cell identity gene *SXI1*α located in the mating type locus. Because *znf1* mutations in several serotype D genetic backgrounds including both mating types did not abolish cell fusion or hyphal formation during **a**-α mating, or filamentation during same sex mating (Lin X and Heitman J, unpublished results), Znf1 is apparently not essential for *Cryptococcus* dimorphic hyphal growth. The *SXI1*α gene is known to be specifically required during **a**-α mating and to be dispensable for filamentation during α-α mating [39], [40] (Lin X and Heitman J, unpublished results); thus this gene does not encode a direct transcription factor effector for the Cpk1 MAPK pathway. Therefore, the remaining candidate is the gene encoding a protein with four N-terminal zinc finger C2H2 domains. Because the mammalian Znf2.2 protein is the closest homolog, this putative zinc finger protein was named *Cryptococcus* Znf2. As is the case for the *MAT2* gene, the *ZNF2* gene is located on a different chromosome (chromosome 8) from the *MAT* locus (chromosome 4). The high level of *ZNF2* expression in XL280α was corroborated by northern blot analysis (Figure S2).

**Table 2:** Genes highly expressed during filamentous growth in XL280α versus in XL34α.

Gene name	Annotation	Fold changes╬	Homolog Accession#
***ZNF1*** **α** *	Zinc finger transcription factor	>10	AAN75722
***MYO2*** **α** *	Microfilament motor	>10	AAN75169
***RPL22*** **α** *	Ribosomal protein	>10	AAN75619
***PNB1***	Para-nitrobenzyl esterase	>10	P37967
***STE3*** **α** *	pheromone receptor	>10	AAF71292
***ZNF2***	Zinc finger, C2H2 type	>10	CAB52138
***PRM1***	Pheromone-regulated multispanning membrane protein involved in membrane fusion during mating	>10	NP_014120
***SPO14*** **α** *	Phospholipase D, catalyzes the hydrolysis of phoshatidylcholine	>10	CAA82103
***RPO41*** **α** *	autophagic vacuole formation-related protein, putative	>10	AAL77196
***DFR1***	Similar to *S. pombe* Dihydrofolate reductase: glycine and purine synthesis, DNA precursor synthesis	>10	YSPDFR1A
***URK1***	Uridine monophosphokinase	>10	P27515
***SCD1***	Unknown, likely involved in establishment and/or maintenance of cell polarity	>10	P40995
***MYO13***	Similar to myosin M	10.0	AAO50967
***UAP1***	Uric acid-Xanthine Permease	8.7	AAN75728
***CPK1***	Mitogen-activated protein kinase (MAPK) involved in mating pheromone response	6.3	NP_009537
***HFM1***	ATP-dependent DNA helicase, meiosis related	5.4	P51979
***UBP14***	Ubiquitin-specific protease that specifically disassembles unanchored ubiquitin chains	4.3	NP_009614
***RUM1*** **α** *	Protein of unknown function	4.3	AAN75714
**183.m01636**	Putative leucoanthocyanidin dioxygenase	4.2	AAB39995
***DIP5***	dicarboxylic amino acid permease	4.0	AAO32605
***PFT1***	Protein farnesyltransferase alpha subunit, putative pheromone maturation-related protein	3.6	P29702
***KIN4***	Serine/threonine-protein kinase	3.6	Q01919
***EBG1***	endo-1,3(4)-beta-glucanase	3.5	AAC17104
***SXI1*** **α** *	Homeodomain protein	3.1	AF542531

*indicates genes encoded by the *C. neoformans* α mating type locus.

**╬:** Average of three replicates of the ratio of the expression level in XL280α and XL34α. For genes expression level that are 10 fold higher, the specific value may not be accurate and thus are indicated by >10 instead.

#GenBank Accession number for the closest homologs.

### Deletions of *MAT2* and *ZNF2* abolish filamentation during a–α and α–α mating

**Figure 1:**
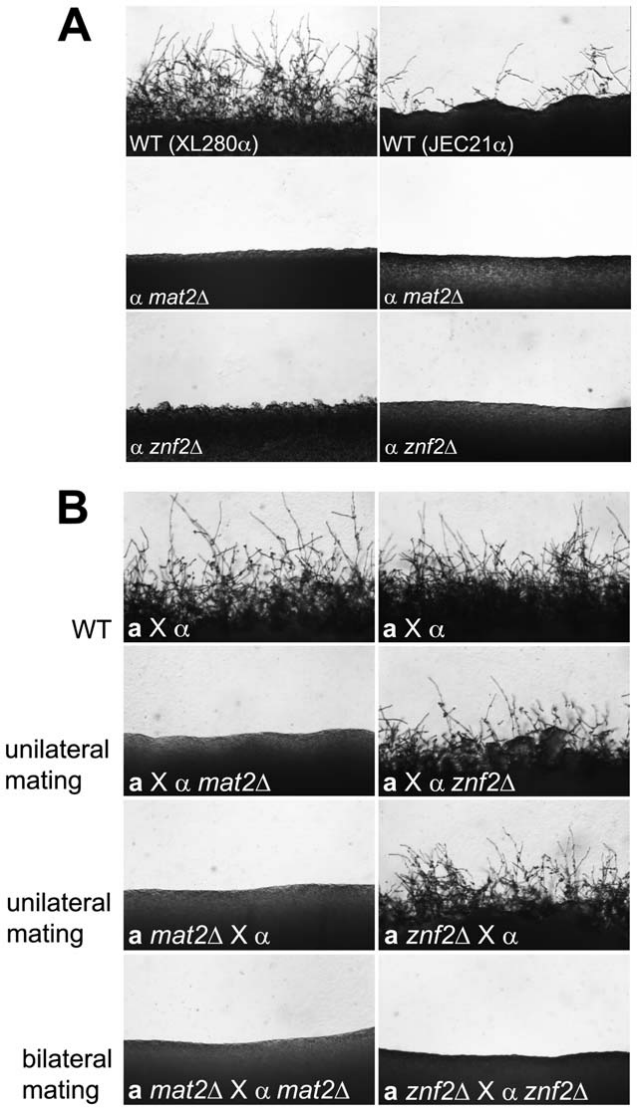
Deletion of either the *MAT2* or the *ZNF2* gene impairs filamentation during a-α and α-α mating. (A) JEC21α, XL280α, and corresponding *mat2*Δ and *znf2*Δ mutants (XL XL926, XL576, XL942, and XL574) were individually incubated on V8 medium for 1 week in the dark at 22°C to examine the ability to differentiate. (B) Appropriate α and **a** mating partners of wild type (JEC21 and JEC20), and *mat2*Δ (XL926 and XL961) and *znf2*Δ (XL576 and XL879) mutants in the JEC21α background were mixed and co-cultured on filamentation agar medium for 48 hours in the dark at 22°C to examine filamentation during unilateral and bilateral matings.

To establish the roles of the *MAT2* and *ZNF2* genes in dimorphic hyphal growth, both genes were deleted via biolistic transformation in the serotype D reference strain JEC21α and strain XL280α backgrounds. In contrast to the hyperfilamentous strain XL280α, which produces filaments surrounding the entire colony periphery on all filamentation inducing media tested, JEC21α only filaments sporadically. Deletion of either the *MAT2* or the *ZNF2* gene blocked filamentation during α-α same sex mating on filament agar (low nitrogen) in both genetic backgrounds (Figure 1A). Similar results were obtained with other filamentation inducing media such as SLAD (low nitrogen) or V8 medium (contains inositol that induces mating [77]) (data not shown).

**a**-α mating of *C. neoformans* is initiated when haploid cells of opposite mating types (**a** and α) fuse with each other and produce dikaryotic hyphae. Because **a**-α mating involves two partners of opposite mating types, both unilateral (a mutant strain crossed with a wild type partner) and bilateral matings (a mutant strain crossed with a mutant partner) for *mat2*Δ and *znf2*Δ mutants were examined to establish their roles in **a**-α mating. No mating hyphae were produced by *mat2Δ* mutants in either unilateral (α *mat2*Δ × **a**, or α × **a** *mat2*Δ) or bilateral matings (α *mat2*Δ × **a** *mat2*Δ) (Figure 1B). This indicates that the presence of a functional *MAT2* gene in both the α and the **a** partner is required for **a**-α mating to occur. Upon prolonged incubation (>2 weeks), unilateral matings occasionally produced a few sporadic mating filaments and thus the block to mating is not absolute (data not shown). A similar phenotype has also been observed in mutants of the Cpk1 MAPK pathway [4]. In contrast, with *znf2Δ* mutants, mating hyphae and basidiospores were produced during unilateral matings (α *znf2*Δ × **a**, or α × **a** *znf2*Δ), albeit at a slightly reduced level compared to matings between wild type partners (α × **a**). However, filamentation was entirely abolished in bilateral matings (α *znf2*Δ × **a** *znf2*Δ) (Figure 1B), even following prolonged incubation. This indicates that *znf2*Δ mutants are bilaterally sterile but the presence of one functional *ZNF2* gene in one of the two partners is sufficient for mating to proceed and Znf2 from either partner can compensate for the absence in the other partner.

### Mat2 is required for cell–cell fusion whereas Znf2 is required for hyphal morphogenesis during mating

**Figure 2:**
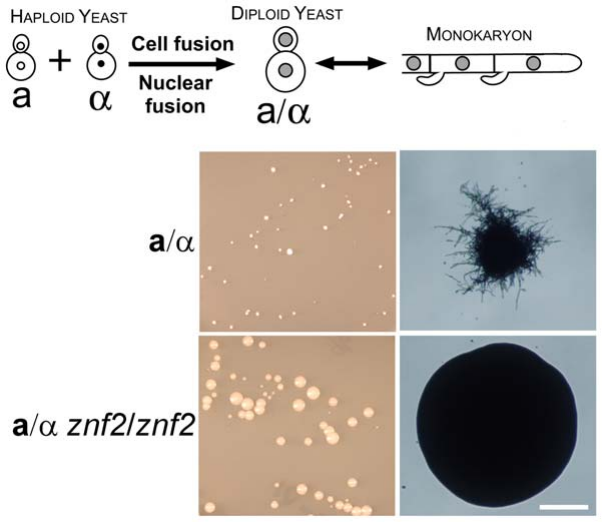
Znf2 is required for hyphal formation after cell-cell fusion during a-α mating. The schematic diagram depicts the mating process of wild-type strains. Haploid α and **a** yeast cells were co-incubated and the cell-cell fusion products, when selected at high temperatures, will undergo nuclear fusion and become heterozygous α/**a** diploid yeast cells. When the environment becomes favorable for filamentation (by lowering the temperature), the α/**a** diploid cells will undergo a morphological transition, produce hyphae, and eventually undergo meiosis and sporulate (not shown here). Appropriately marked α and **a** strains of wild type (XL877 and XL878) and *znf2*Δ mutants (XL874 and XL875) were paired, mixed, and co-cultured on V8 medium for 15 hours at room temperature in the dark. The cocultures were collected and transferred to YNB minimal medium to select for fusion products as shown in the left panel. Microscopic images of the colony derived from the fusion event are shown in the right panel. Scale bar, 200 micrometers.

**a**-α mating involves several steps: cell fusion between **a** and α partners, dikaryotic hyphae formation, basidium formation, and sporulation. Because mutation of either *MAT2* or *ZNF2* blocked hyphal formation during bilateral matings, these two genes could be critical for cell-cell fusion, the initiation of hyphal morphogenesis, or both. To define the step(s) in α-**a** mating during which Mat2 and Znf2 function, their roles in cell fusion were examined. Yeast cells of genetically marked wild type α and **a** strains, α *mat2*Δ and **a** *mat2*Δ mutants, and α *znf2*Δ and **a** *znf2*Δ mutants were paired and incubated on V8 agar for 15 hours in the dark at room temperature. The cocultured cells were collected and plated on appropriate media to select for fusion products (see “Materials and Methods” for details)(Figure 2). No fusion products were detected from the bilateral pairing of α *mat2*Δ and **a** *mat2*Δ mutants. We further tested the efficiency of cell fusion from the unilateral pairing of a wild type partner and *mat2*Δ mutants (α *mat2*Δ + **a** or α + **a** *mat2*Δ) under the same experimental conditions. Again, no cell fusion products were recovered. These observations indicate that Mat2 is required for cell-cell fusion events and both partners must carry a functional *MAT2* gene in order for cell fusion to occur. This phenotype is again reminiscent of that of Cpk1 MAPK cascade mutants [4].

In contrast, the bilateral pairing of α *znf2*Δ and **a** *znf2*Δ mutants yielded 600% more fusion products than the control, indicating that Znf2 is not necessary for cell fusion, and may normally repress cell fusion during α-**a** matings. Interestingly, while the fused wild type diploids (**a**/α) produced very small colonies and then readily filamented at ambient temperatures (Figure 2), the fused mutant **a**/α diploids (*znf2*Δ/*znf2*Δ) derived from XL875α (*znf2*::*NAT^r^ ade2*) and XL874**a** (*znf2*::*NAT^r^ lys1*) mutants did not produce any filaments and continued growing as budding yeast, yielding large yeast colonies under the same conditions (Figure 2). This result indicates that Znf2 is critical for hyphal formation and functions after the cell-cell fusion event during **a**-α mating. As formation of conjugation tubes is necessary for cell-cell fusion during bisexual mating [27], the observation that *znf2Δ* cells can undergo cell fusion successfully but are unable to form hyphae suggests that the mechanisms to produce conjugation tubes are not identical with those to produce true hyphae.

To determine if Znf2 also functions after the cell-cell fusion event during same sex mating (α-α mating), mutant α/α diploids (*znf2*Δ/*znf2*Δ) were generated by fusion between two auxotrophically marked α mutants XL872α (*znf2*::*NAT^r^ ade2 lys1*) and XL873α (*znf2*::*NAT^r^ ura5*). The α/α homozygous diploid mutant (*znf2*Δ/*znf2*Δ) again only grew in the yeast form (data not shown), similar to the **a**/α heterozygous diploid mutant (*znf2*Δ/*znf2*Δ) described above. Taken together, the key role of Znf2 is to enable or support hyphal morphogenesis after cell fusion.

### Mat2 regulates pheromone sensing and response, whereas Znf2 does not

**Figure 3:**
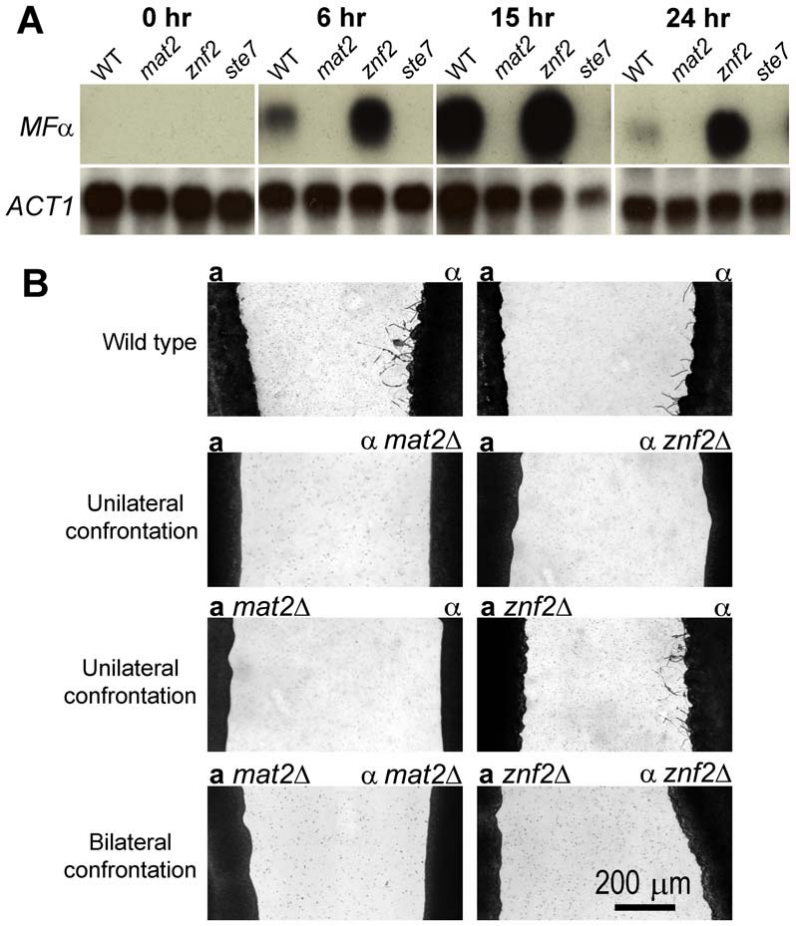
Mat2 is required for pheromone sensing and production, whereas Znf2 is dispensable for the response during a-α mating. (A) Northern blot analysis of the expression pattern of the *MF*α gene during **a**-α mating in wild type, *mat2*Δ, *znf2*Δ, and *ste7*Δ mutants in JEC21 background at 0 hours, 6 hours, 15 hours, and 24 hours post coinoculation of the **a** and α mating partners. The expression level of the actin gene (*ACT1*) serves as a control. (B) Confrontation assays of the effect of *mat2* and *znf2* mutations on the ability of **a** and α cells to produce and respond to pheromones. Scale bar, 200 micrometers.

The Cpk1 MAPK pathway mediates pheromone sensing and response during mating [4]. To determine whether Mat2 and Znf2 are important for pheromone production during mating, transcription of the *MF*α pheromone gene in wild type, *mat2*Δ, *znf2*Δ, and *ste7*Δ mutants was monitored during bilateral **a**-α matings. The *ste7*Δ mutants were chosen as representative of the Cpk1 MAPK cascade as Ste7 is the MEK upstream of the MAPK Cpk1, and also because this gene was identified in our insertional mutagenesis screen (Table 1). As shown in Figure 3A, no pheromone transcript was detected in the wild type prior to the initiation of mating, but its level increased at 6 hours post-coincubation, reached the highest level at 15 hours post-coincubation, and returned to a lower basal level by 24 hours. A similar pheromone expression pattern of wild type cells during mating has been observed independently (Griffith B, Fraser J, and Heitman J, unpublished results). In contrast, no *MF*α transcript was detected in either the *ste7*Δ or the *mat2*Δ mutants over the time course studied, indicating that Mat2 and Ste7 are required for pheromone induction. This phenotype of the *mat2*Δ and the *ste7*Δ mutants is in accord with the known roles of the Cpk1 MAPK pathway in pheromone sensing and response. *MF*α expression in the *znf2*Δ mutant followed a pattern similar to wild type, with an increased level of pheromone production during early stages of mating followed by reduced levels. Interestingly, at all time points examined (except at the zero time point at which no pheromone expression was observed), a higher level of *MF*α was observed in the *znf2*Δ mutant. The elevated level of pheromone expression during bilateral matings in the *znf2*Δ mutants may be responsible for the increased efficiency in cell fusion observed. Taken together, our evidence indicates that Mat2 likely functions as a direct target of the Cpk1 MAPK pathway, whereas Znf2 functions further downstream.

Confrontation assays were performed to assess the effect of *mat2* and *znf2* mutations on the ability of **a** and α cells to produce and respond to pheromones. When **a** and α cells are grown in close proximity but not mixed, α cells produce conjugation tubes or monokaryotic hyphae in response to **a** cells/MF**a** pheromone, whereas **a** cells predominately become enlarged in response to α cells/MFα pheromone [59]. In addition to inducing **a** cell responses, MFα pheromone also provides a positive autocrine feedback to the pheromone response pathway in α cells to promote cell fusion [78]. As shown in Figure 3B, **a** *mat2*Δ cells failed to induce wild type α cells to produce conjugation tubes or filaments, indicative of defects in pheromone production. The α *mat2*Δ cells did not produce any filaments when confronted with wild type **a** cells, reflecting defects also in response to pheromone produced by the confronting mating partner. The phenotype of *mat2*Δ mutants was identical to that of *ste7*Δ mutants (Figure 3B). These observations are consistent with the northern blot analysis showing no detectable *MF*α pheromone gene expression during bilateral matings of *mat2*Δ or *ste7*Δ mutants and are also consistent with previous findings about the critical roles of components of the Cpk1 MAPK pathway in pheromone production and response [4]. In contrast, the **a** *znf2*Δ mutant cells successfully induced wild type α cells to produce filaments, indicating no defects in pheromone production. However, the α *znf2*Δ cells did not produce any conjugation tubes or hyphae when confronted with wild type **a** cells. Based on the northern blot analysis and cell fusion assays, α *znf2*Δ cells have no defect in producing or responding to pheromone. Thus, the defect of the *znf2*Δ mutant likely reflects an inability to form hyphae. Again, these lines of evidence support the hypothesis that Mat2 functions as the direct transcription factor effector of the Cpk1 MAPK pathway, whereas Znf2 is a more terminal morphology determinant.

To examine the epistatic relationship between *ZNF2* and the Cpk1 MAPK pathway, a *znf2*Δ *cpk1*Δ double mutant was isolated following a genetic cross between the *znf2*Δ mutant and the *cpk1*Δ mutant. This double mutant (XL1131α) showed severe impaired unilateral mating when coincubated with the wild type mating partner JEC20**a** (data not shown), similar to the *cpk1*Δ single mutant [4]. This observation again suggests that Znf2 functions downstream of Cpk1.

**Figure 4:**
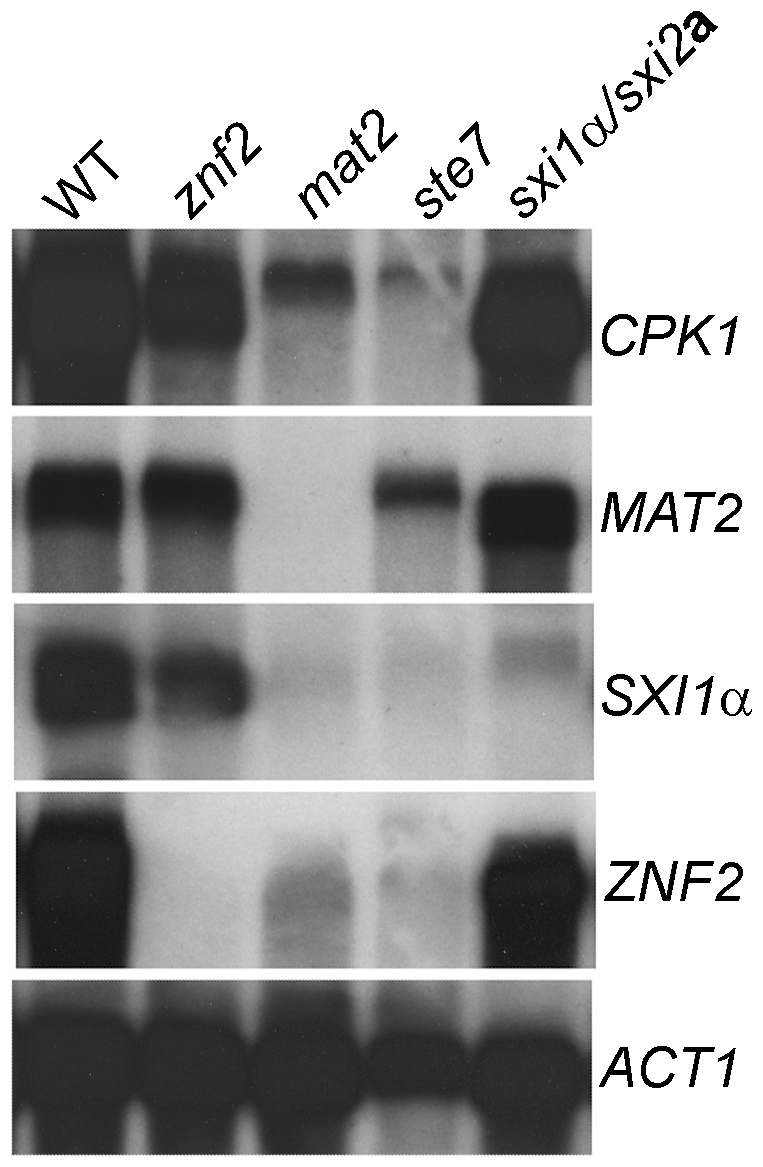
Znf2 and the Sxi1α/2a complex do not regulate Mat2 or the Cpk1 pathway at the transcript level. The expression pattern of the *CPK1*, *MAT2*, *SXI1*α, and *ZNF2* genes during bilateral matings in the following strain pairs (α × **a**, α *znf2*Δ × **a** *znf2*Δ, α *mat2*Δ × **a** *mat2*Δ, α *ste7*Δ × **a** *ste7*Δ, and *sxi1*αΔ × *sxi2* **a**Δ in JEC21 background) that had been cocultured on V8 medium (pH = 7.0) for 24 hr.

To further test the relationship between Mat2, Znf2, the Cpk1 pathway, and the Sxi1α/2**a** complex, we examined the expression pattern of the *CPK1*, *MAT2*, *SXI1*α, and *ZNF2* genes during bilateral matings in the following strain pairs: α × **a**, α *znf2*Δ × **a** *znf2*Δ, α *mat2*Δ × **a** *mat2*Δ, α *ste7*Δ × **a** *ste7*Δ, and *sxi1*αΔ × *sxi2* **a**Δ, which were cocultured on V8 medium (pH 7.0) for 24 hr. As shown in Figure 4, minimal or no *ZNF2* or *SXI1*α transcripts were detectable in the *ste7*Δ and *mat2*Δ mutants, indicating that the Cpk1 MAPK pathway regulates the transcription of *ZNF2* and *SXI1*α. The transcription level of the *CPK1* gene was downregulated in the *mat2*Δ and possibly also in *ste7*Δ mutants, suggesting that mutations in the Cpk1 MAPK pathway may reduce transcription of other components in the pathway, consistent with positive feedback regulation of the pheromone sensing pathway. The expression levels of *SXI1*α and possibly *CPK1* were slightly reduced in the *znf2*Δ mutants, suggesting that *ZNF2* may also normally positively regulate the expression of these genes. On the other hand, the transcription levels of *MAT2* and *CPK1* were not affected in the *sxi1*αΔ/*sxi2* **a**Δ mutants, suggesting that the Sxi1α/Sxi2**a** complex does not regulate the Cpk1 MAPK pathway. In addition, no significant change in the transcription level of *ZNF2* was observed in the *sxi1*αΔ/*sxi2* **a**Δ mutants, indicating that the Sxi1α/Sxi2**a** complex does not regulate Znf2 at the transcriptional level. Similar gene expression results in these mutants during bilateral matings were also observed in the genome wide expression studies (Tables S1, S2, S3). Again, these gene expression results support the conclusion that Mat2 is part of the Cpk1 MAPK pathway and together they regulate the expression of *ZNF2* and *SXI1*α/2**a** during mating. Znf2 also may play a modest role in regulating the transcription of *CPK1* and *SXI1*α. In contrast, deletion of *SXI1*α shows no effect on the transcription the MAPK pathway or *ZNF2*.

### Znf2 evokes hyphal formation, whereas Mat2 is dispensable

As both Mat2 and Znf2 are required for hyphal growth during **a**-α and α-α mating, it was not evident whether abolished hyphal growth in the *mat2*Δ and *znf2*Δ mutants was attributable to their role in hyphal morphogenesis *per se* or to an involvement in relaying signals to stimulate the initiation of hyphal formation. For example, the homeodomain cell identity protein complex Sxi1α/Sxi2**a** initiates hyphal growth after the cell-cell fusion event during **a**-α mating [39], [40], but neither Sxi1α nor Sxi2**a** is required for formation of hyphae as *sxi1*αΔ or *sxi2* **a**Δ mutants can still filament during same sex mating [39], [40] (Lin X and Heitman J, unpublished results). Thus, the Sxi1α/Sxi2**a** complex stimulates mating hyphal formation [39] by relaying stimuli, but is not required for hyphal morphogenesis *per se*.

**Figure 5:**
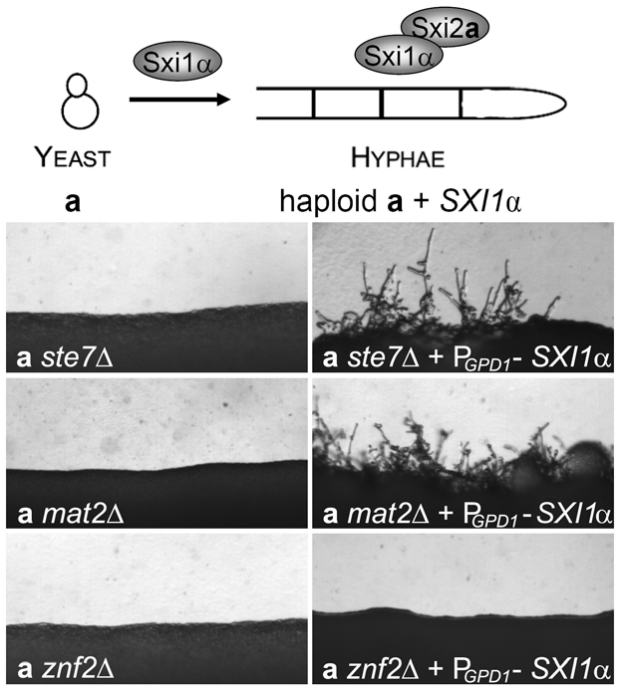
Mat2 and the Cpk1 MAPK cascade are not required for hyphal morphogenesis whereas Znf2 is a hyphal morphology determinant. The schematic diagram shows that if haploid **a** cells that harbor the *SXI2* **a** gene in their genome are transformed with the *SXI1*α gene (indicated by the Sxi1α above the arrow), both the *SXI1*α and *SXI2* **a** genes will be expressed in the **a** cells. The protein complex formed by these two cell-identity proteins renders the haploid **a** cells competent to produce hyphae like **a**/α diploid or **a**-α dikaryons without the requirement for the fusion of α and **a** cells. The **a** cells with *ste7*Δ, *mat2*Δ, or *znf2*Δ mutation in JEC21 background and the corresponding mutants bearing the P*_GPD1_*-*SXI1*α transgene were cultured on filamentation agar medium and incubated in the dark for 48 hours at 22°C.

Because Mat2 is required for cell-cell fusion, examining its role in hyphal formation during mating after the cell-cell fusion step is challenging. To circumvent the cell-cell fusion step, we decided to take advantage of the previous observation that haploid cells carrying the Sxi1α/Sxi2**a** complex mimic **a**/α diploids or **a**-α dikaryotic cells and can filament in response to temperature and environmental cues [39], [40], [69], [79] (Figure 5, top panel). Transforming **a** cells with the α cell identity gene *SXI1*α, or α cells with the **a** cell identity gene *SXI2* **a** generates haploids with the cell-identity complex derived from **a** and α cells. Here we transformed mutants of **a** mating type with the *SXI1*α gene under the control of the constitutively active *GPD1* promoter. As a control, the presence of P*_GPD1_*-*SXI1*α in *ste7*Δ, *mat2*Δ, or *znf2*Δ mutants in the α cell type did not stimulate filament production (data not shown). As shown in Figure 5, transforming the **a** *ste7*Δ mutant with P*_GPD1_*-*SXI1*α enabled the originally non-filamentous **a** cells to produce hyphae (wild type JEC20**a** does not produce hyphae). Similarly, the presence of the P*_GPD1_*-*SXI1*α gene in **a** *mat2*Δ cells also stimulated formation of hyphae (Figure 5). In contrast, no filaments were observed when P_GPD1_-*SXI1*α was transformed into the **a** *znf2*Δ cells (Figure 5).

These observations indicate that Mat2, like Ste7, is not necessary for hyphal morphogenesis *per se* but is required for relaying the pheromone stimulus to initiate hyphal formation. Conversely, Znf2 is not required for sensing pheromone cues that stimulate hyphal growth, but it is a terminal determinant of hyphal morphogenesis. That said, Znf2 does influence pheromone sensing and cell fusion as the cell fusion efficiency and the pheromone transcript level are higher in the *znf2Δ* mutants compared to the wild type control. However, these differences caused by *ZNF2* deletion are quantitative instead of qualitative. In contrast, its function is hyphal growth is absolutely required.

### Mat2 is a key element of the Cpk1 MAPK pathway, while Znf2 functions in distinct but overlapping pathways

**Figure 6:**
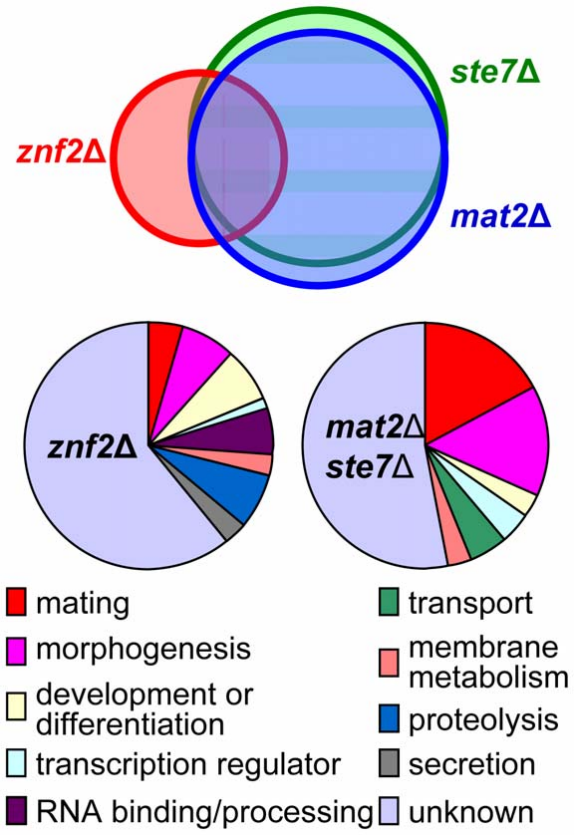
Gene expression profiles of *ste7*Δ, *mat2*Δ, and *znf2*Δ mutants during bilateral mating. The top panel shows the nearly identical profiles between *ste7*Δ and *mat2*Δ mutants with 96% overlap in contrast to only 47% congruence with genes differentially expressed in *znf2*Δ mutants. The bottom panel shows a classification of the genes that were differentially expressed in the mutants based on GO ontology.

Genes transcriptionally regulated by the two novel transcription factors were examined at the whole genome level employing the *C. neoformans* genome wide 70 mer oligonuleotide microarray based on the JEC21α genome sequence. Transcripts produced by *ste7*Δ, *mat2*Δ, and *znf2*Δ mutants of both mating types in the JEC21 (α)/JEC20(**a**) backgrounds during bilateral matings after 15 hours of co-incubation on V8 medium were compared to those produced by the wild type (JEC21 × JEC20). Genes differentially expressed in the *mat2Δ* mutants under this condition are almost identical with those in the *ste7Δ* mutants (Figure 6)(Table S1). Discrepancies in a few genes might be caused by variations in of the microarray experiments. This result further supports the conclusion that Mat2 is a direct downstream transcription factor of the Cpk1 MAPK pathway. Many down regulated genes encode products known to be involved in mating, including those involved in pheromone synthesis and processing, pheromone receptors, G proteins upstream of the MAPK pathway, the MAP kinase Cpk1, and the homodomain protein Sxi1α (Figure 6)(Table S1). Down-regulation of these genes is consistent with the northern hybridization analysis presented earlier. Genes involved in morphogenesis were the second largest group that showed transcriptional differences compared to wild type, including several genes in the septin family and other cell cytoskeletal genes (cell wall components). Genes likely involved in transport, transcription, or development were also identified.

In contrast, fewer genes were found to be differentially expressed in the *znf2*Δ mutants during bilateral mating (Figure 6)(Table S2). The most striking difference in the expression profile of *znf2*Δ mutants compared to those of the *ste7*Δ or the *mat2*Δ mutants was that genes involved in the pheromone sensing and response were not significantly differently expressed in the *znf2*Δ mutants when compared to the wild type (Table S2). Additionally, genes involved in cellular transport were not significantly changed, and fewer genes involved in morphogenesis were identified. This observation supports the conclusion that Znf2 does not regulate the pheromone sensing pathway and functions as a more terminal hyphal morphogenesis determinant. It is interesting to note that more genes functioning in RNA binding or processing, protease activities, and secretion were differentially expressed in the *znf2*Δ mutant compared to the *ste7*Δ or the *mat2*Δ mutants. There are many uncharacterized genes with no defined homologs, and these genes cannot at present to be assigned to any specific process.

To examine the expression profiles of these mutants during unisexual mating, transcripts produced by *ste7*Δ, *mat2*Δ, and *znf2*Δ mutants in the XL280(α) background during unisexual mating after 24 hours of incubation on V8 medium were compared to those produced by the wild type strain XL280. Although different numbers of genes were selected compared to the those described above due to the difference in quality of the microarray data, about 48% of the genes that were selected for differential expression in the *ste7* mutant during unisexual mating in the XL280 background were identical to those selected in the α *ste7* × **a** *ste7* bilateral mating in the JEC21/JEC20 background (highlighted in Table S3). Similarly, 42% of the genes for the *mat2* mutant and 48% for the *znf2* mutant during unisexual mating in the XL280 background were identical to those selected in the α *mat2* × **a** *mat2* and α *znf2* × **a** *znf2* bilateral matings in the JEC21/JEC20 background respectively (Table S3). Again, genes involved in the pheromone response pathway were not significantly differently expressed in the *znf2*Δ mutant but were in the *ste7*Δ and *mat2*Δ mutants. Genes that were dramatically suppressed in the mutants compared to the corresponding wild type controls tend to be consistently represented under both conditions.

### Mat2 is dispensable for virulence, while Znf2 is a negative regulator of pathogenicity

**Figure 7:**
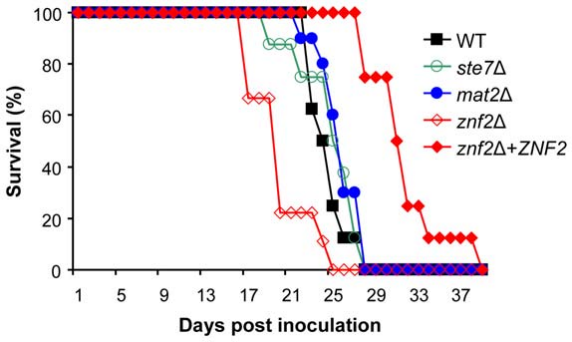
Mat2 is dispensable for virulence, while Znf2 is a negative regulator of pathogenicity. Animals (10 each group) were intranasally infected with 5×10^4^ yeast cells of the wild type (H99), *ste7*Δ (YSB345), *mat2*Δ (XL1598), *znf2*Δ (XL1601), and *znf2*Δ-*ZNF2* (XL1643) strains. The survival rates of animals were plotted against time after inoculation, and *P* values compared to the wild type control are: *ste7*Δ (*P* = 0.68), *mat2*Δ (*P* = 0.048), *znf2*Δ (*P* = 0.0026), *znf2*Δ+*ZNF2* (*P* = 0.00034).

Dimorphic transitions between yeast and hyphae have been linked to virulence in several pathogenic fungi. To determine if Mat2 and Znf2 are necessary for *Cryptococcus* pathogenicity, the impact of gene deletions on *Cryptococcus* virulence potential was examined in a murine inhalation model of cryptococcosis. Because serotype A is in general more prevalent and more virulent than serotype D [80]–[82], the roles of Mat2 and Znf2 in virulence were examined in the highly virulent serotype A H99 background. Disruption of the *MAT2* and the *ZNF2* genes in strain H99 caused similar defects during **a**-α mating as observed in serotype D backgrounds (data not shown), indicating conserved functions of these two proteins in the two serotypes. Because Mat2 is required for cell fusion and the *mat2*Δ mutant is unable to complete unilateral mating, the linkage between the mutation and the phenotype was only confirmed for *znf2*Δ via genetic crosses (data not shown). Introducing a wild type copy of *ZNF2* ectopically into the *znf2*Δ mutant restored the mating defect, indicating that the phenotype was indeed caused by mutation in the *ZNF2* gene (data not shown). To assess the impact of the deletion of *MAT2* or *ZNF2* on *Cryptococcus* virulence potential, animals were intranasally infected with yeast cells of the deletion mutants (*ste7*Δ, *mat2*Δ, and *znf2*Δ) and wild type H99 and their survival was monitored. As shown in Figure 7, the *ste7*Δ and *mat2*Δ mutants were of equivalent virulence compared to the wild type control. This result is consistent with previous observations that components of the Cpk1 MAPK pathway are dispensable for *Cryptococcus* virulence in murine models of cryptococcosis [4]. That disruption of *MAT2* exerted no or minimal effect on virulence is in accord with the model that Mat2 is a downstream transcription factor of the Cpk1 MAPK pathway.

In contrast, a modestly enhanced virulence was observed for the *znf2Δ* mutant and the difference from the wild type is statistically significant (Figure 7A). This virulence experiment was repeated with a modestly higher infectious dose and a similar pattern was observed (Figure S3). The complemented strain, in which the *ZNF2* gene with its native promoter and terminator was ectopically integrated into the genome, was modestly less virulent than the wild type strain. Because the integration site of the wild type copy of *ZNF2* occurs in an intergenic region (data not shown), it is unlikely that other genes regulating virulence were affected. The observation that the introduced wild type copy of *ZNF2* restored the mating ability of the *znf2*Δ mutant suggests that the reduced virulence in this strain is likely due to a position effect, which may increase the activity of Znf2. This result suggests that Znf2 acts as a negative regulator of virulence and that there might be an inverse relationship between *ZNF2* activity and virulence potential. This hypothesis warrants additional testing in future investigations.

### Mat2 and Znf2 do not regulate defined virulence traits *in vitro*

To determine if the virulence potential of the *mat2*Δ and *znf2*Δ mutants correlates with their *in vitro* phenotypes with respect to well-characterized virulence traits of *Cryptococcus*, the mutants and control wild type strains were assayed for melanization, capsule production, and the ability to grow at mammalian body temperature 37°C. Disruption of *MAT2* or *ZNF2* did not cause any apparent alteration in these traits in all of the backgrounds tested (Figure S4). Our results indicate that Znf2 regulates *Cryptococcus* virulence via means other than the well-defined characterized virulence factors examined.

## Discussion

**Figure 8:**
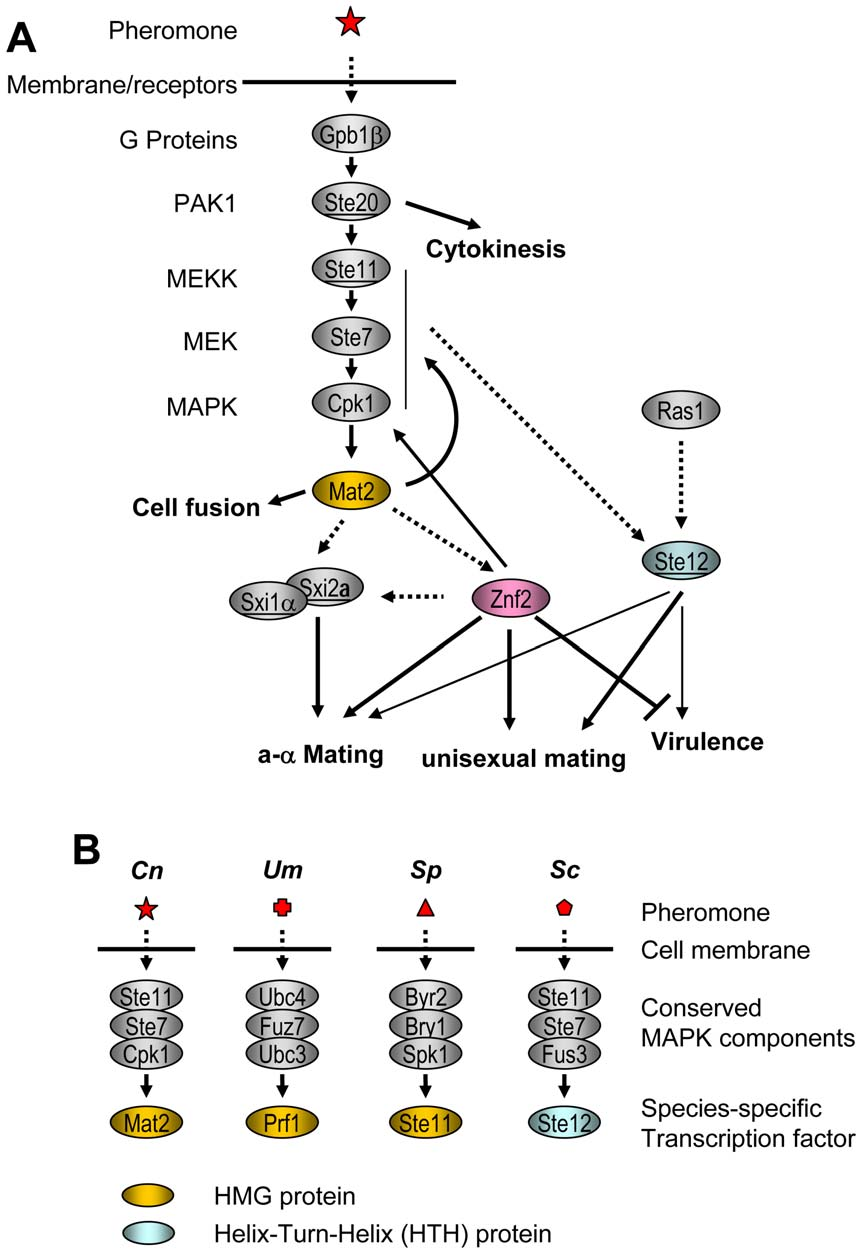
Mat2 and Znf2 operate cellular circuits orchestrating opposite- and same-sex mating in *C. neoformans*. (A) Mat2 is depicted as the direct target of the MAPK pathway and it further serves to regulate the homodomain complex and Znf2. Genes encoded by the *MAT* locus are underlined. The dotted arrow indicates predicted interactions. (B) HMG domain proteins serve as the transcription factor downstream of the MAPK in the pheromone sensing pathways of divergent fungal species. *Cn*: *C. neoformans*. *Um*: *U. maydis*. *Sp*: *S. pombe*. *Sc*: *S. cerevisiae*.

Several lines of evidence presented in this study converge to implicate Mat2, an HMG transcription factor, and Znf2, a zinc finger transcription factor, as central components governing opposite and same sex mating in *C. neoformans*. The congruence of the transcriptional profiles of *ste7*Δ and *mat2*Δ mutants and their parallel phenotypes *in vitro* and *in vivo* provide evidence that these two proteins function in a common pathway. Based on previous observations that HMG proteins serve as the downstream transcription factors in the mating pathway in diverse fungal species [72], [83] (Figure 8B), we hypothesize that Mat2 is a direct target of Cpk1 in *Cryptococcus* (Figure 8). The well-known transcription factor Ste12 is unlikely to be a direct major downstream target of Cpk1 based on the observations that deletion of *STE12* only modestly reduces but does not abolish either opposite sex mating or same-sex mating (Figures S1, S5). However, a direct interaction between Cpk1 and Mat2 has yet to be confirmed. Similarly, the relationship between the MAPK pathway/Mat2 and Ste12 remains to be illustrated.

In comparison, our evidence indicates that Znf2 is required for hyphal morphogenesis during both unisexual mating and bisexual mating, and is not critical for the pheromone response (Figure 8A). Znf2 may regulate the cell identity homeodomain protein Sxi1α at the transcriptional level during mating.

In dimorphic fungi, the link between dimorphism and virulence has been recognized for decades and is under intensive investigation [6]–[19]. *C. neoformans*, on the other hand, has been typically considered as a yeast [6]. In addition, because the two central pathways involved in mating dimorphic hyphal growth (the Cpk1 MAPK pathway and the Sxi1α/Sxi2**a** complex) have been shown to have no or minimal impact on virulence, the association between dimorphism and virulence in *C. neoformans* has not been a focus of research efforts [6]. However, based on the evidence adduced in this study, we submit that the impaired dimorphic mating hyphal growth in the mutants of the Cpk1 MAPK pathway or the Sxi1α/Sxi2**a** complex may be attributable to defects in sensing extra-cellular or intra-cellular cues that stimulate initiation of filamentation rather than impaired ability to undergo hyphal growth. For example, disruption of the *SXI1*α gene affects **a**-α mating hyphal growth but filamentation during same sex mating still occurs [40]. Here we show that disruption of *STE7* and *MAT2* markedly impairs hyphal growth during both **a**-α and α-α mating, but with the activation of Sxi1α/Sxi2**a**, hyphal growth can be partially restored (Figure 5). Studies in *C. albicans* and *U. maydis* have also shown that disruption of the pheromone-sensing pathway results in defective hyphal growth under some but not all conditions [18], [84]–[88]. These studies echo the same message that mutations in signal transduction pathways do not abrogate the inherent ability of a strain to undergo hyphal growth. If it is the ultimate inherent ability to undergo dimorphic morphogenesis, not relay the signal, that affects virulence in *C. neoformans*, then it is not surprising that Mat2, like other components in the Cpk1 MAPK signal transduction pathway or the Sxi1α/Sxi2**a** complex, is dispensable for virulence.

Because *Cryptococcus* hyphae are rarely observed in human or animal tissues [37], [89]–[94], and the host conditions suppress hyphal growth, it is possible that cells locked in the yeast form, either naturally or by genetic manipulation (e.g. deletion of *ZNF2*), will show enhanced virulence in animal models of cryptococcosis, whereas cells locked in a filamentous form would be avirulent. This hypothesis is supported by previous observations that the filamentous form shows drastically reduced pathogenicity in animal models when used as the infectious inoculum [6], [95]–[98] and the filamentous form can immunize animals against subsequent challenge of virulent *C. neoformans* in the yeast form [99]–[101]. Thus, studies on cell shape and virulence in this organism could have significant clinical impact. However, the exact molecular mechanisms by which Znf2 determines the dimorphic transition to hyphal form and affects virulence potential remain elusive and may reflect distinct processes that Znf2 regulates. Based on the gene expression studies of the *znf2Δ* mutants during mating (Figure 6 and Tables S2, S3), Znf2 may regulate (1) lysis of protein or carbohydrate, (2) RNA processing, (3) tracking, or (4) lipid metabolism. Identification and investigation of the direct targets of this transcription factor will be essential to understand the functions of Znf2 in morphogenesis and virulence, and will be the focus of our future research.

The recent discovery that *C. albicans* can also undergo same sex mating underscores the importance of transitions in modes of sexual reproduction in pathogenic microbes [42], [102]. That two divergent human pathogens both underwent independent transitions enabling same sex mating to occur illustrates the plasticity of sexual reproduction and its contribution to their evolution. Given that same sex mating arose independently in these two divergent human fungal pathogens suggests that other examples likely remain to be discovered in the fungal kingdom, and possibly also in other more divergent eukaryotes such as pathogenic parasites [103].

The ultimate questions of how unisexual and bisexual reproduction evolved and how they are regulated in *C. neoformans* remain to be answered. Previous studies on the genetic circuits controlling mating in *S. cerevisiae* may prove illustrative. The **a**/α diploid *S. cerevisiae* cell type, instructed by the cell identity **a**1/α2 complex, is competent to undergo meiosis and produce meiotic progeny. By contrast, **a**/**a** and α/α diploid yeast cells are normally incompetent to undergo meiosis. However, mutations in the meiosis repressor gene *RME1* can bypass the normal requirement for **a**1/α2 and enable **a**/**a** *rme1*/*rme1* and α/α *rme1*/*rme1* mutants to undergo meiosis at a lower efficiency [104]–[107]. Thus, mutation of a single gene bypasses the normal requirement for contributions from the two mating type alleles in the genetic circuit that enables diploid *S. cerevisiae* cells to engage in sexual reproduction. Analogous genetic changes in the regulatory circuit that normally orchestrates opposite sex mating of *C. neoformans* may have enabled the evolution of modified circuits enabling same sex mating, and in so doing bypassed the normal requirement for either Sxi1α or Sxi2**a**.

The nature of signaling pathways and differentiation cascades as linear pathways vs. branched networks may influence the developmental plasticity of the evolution of morphogenesis and sexual reproduction. We hypothesize that a branched network governs sexual reproduction of *C. neoformans* and that same sex mating may employ some of the components in the circuits governing opposite sex mating. In essence, mutations and epistatic interactions arose that allowed the cell to bypass the normal requirement for Sxi1α/Sxi2**a** for sexual reproduction, and other parallel (MAPK/Mat2 or Ste12) or downstream morphogenesis determinants (Znf2), subsumed a central essential role indicating cell fate and the ability to undergo sexual reproduction (Figure 8).

## Materials and Methods

### Ethics statement

All the animal work was performed according to the guidelines of NIH and Duke University Institutional Animal Care and Use Committee (IACUC).

### Strains and growth conditions

Strains used in this study and their sources are listed in Table S4. Cells were maintained on rich YPD (1% yeast extract, 2% BactoPeptone, and 2% dextrose) or minimum YNB medium (Yeast Nitrogen Base Medium, Difco, Detroit, MI) media. For marker screening, YPD+NAT and YPD+NEO media were employed for strains with dominant drug resistance markers while synthetic complete medium minus adenine (SC-ade), uracil (SC-ura), or lysine (SC-lys) were utilized for strains with auxotrophic markers. Mating and cell fusion assays were conducted on V8 solid medium (pH = 7.0 or pH = 5.0) [108] in the dark at 22°C. Filamentation agar [34] or synthetic low ammonium dextrose (SLAD) agar (YNB without amino acids plus 50 µM ammonium sulfate) were used for monokaryotic fruiting and confrontation assays.

### Generation of insertional mutants and screening for mutants defective in filamentation

Insertional mutagenesis via *Agrobacterium* mediated transformation in XL280α was performed essentially as described previously [68]. Briefly, *A. tumefaciens* strain LBA4404 containing a pPZP-NATcc plasmid [70] was grown for 48 h at 25°C in Luria-Bertani medium with kanamycin in shaking cultures. Bacterial cells were washed twice with sterile water and suspended in induction medium with 100 µM acetosyringone (600 nm of 0.15) and incubated for another 6 hours. *C. neoformans* XL280 cells grown overnight in YPD were washed in induction medium and resuspended at 10^7^ cells/ml. Equal aliquots (200 µl) of *C. neoformans* and *A. tumefaciens* were mixed, plated onto induction medium agar, and incubated at room temperature for 3 days. The cells were scraped from the plates and transferred to YPD medium with nourseothricin (NAT) and cefotaxime (each at 100 µg/ml). 3600 transformants were examined for filamentation on V8 juice solid medium (pH = 7.0) after incubation for 7 days at room temperature in the dark. Mutants that exhibited no hyphal growth were selected. To identify the insertion sites in selected mutants, genomic DNA was digested with a restriction enzyme, purified, and self-ligated. PCR amplicons from the ligation using primers AI076/AI077 were sequenced as described previously [68], [70]. Sequences of the flanking regions were used to BLAST the *C. neoformans* serotype D JEC21 genome database to determine the T-DNA insertion sites and subsequently the mutated genetic loci.

### RNA purification and microarray analysis

For the initial search for transcription factors, total RNA was purified from strains XL280 and XL34 after incubation on V8 medium (pH 7.0) at room temperature for 24 hours using TRIzol Reagent according to the manufacturer's instructions (Invitrogen). Cy3 and Cy5 -labeled cDNA was generated by incorporating amino-allyl-dUTP during reverse transcription of 10 µg of total RNA as described previously [76] and competitively hybridized to a partial genome array generated previously in the Heitman laboratory [76]. After hybridization, arrays were scanned with a GenePix 4000B scanner (Axon Instruments, http://www.axon.com) and analyzed by using GenePix Pro version 4.0 and BRB array tools (developed by Richard Simon and Amy Peng Lam at the National Cancer Institute; http://linus.nci.nih.gov/BRB-ArrayTools.html) as described previously [38], [109].

For later transcriptional profiling of various mutants, total RNA was extracted from bilateral matings (α × **a**, α *ste7*Δ × **a** *ste7*Δ, α *mat2*Δ × **a** *mat2*Δ, and α *znf2*Δ × **a** *znf2*Δ) after incubation on V8 medium (pH = 7.0) at 22°C for the indicated time. They were either processed for Northern Blot analysis or for the whole genome microarray analysis as described above except that they were hybridized to a *C. neoformans* whole genome 70 mer oligo array with additional serotype-specific and mating-type–specific 70 mer oligos for genes in the *MAT* locus designed by Brian Griffith and James Fraser in the Heitman lab [38], [109]. Information about the array can be found at the following Web site: http://genome.wustl.edu/services/microarray/cryptococcus_neoformans). The array was analyzed similarly as described above.

### Northern blots

RNA was separated on agarose gels blotted to nylon membrane. Redi-Prime II kit (Amersham) was used to generate probes. For MFα pheromone gene expression analysis, total RNA was extracted from cocultures of mating partners on V8 medium (pH = 7.0) at 0, 6 hr, 15 hr, and 24 hr after incubation in the dark at room temperature. The *C. neoformans* actin gene transcript served as a control. For the time course examination of MFα expression level, total RNA was used. For the examination of expression pattern of the *CPK1*, *MAT2*, *SXI1*α, and *ZNF2* genes during bilateral matings, mRNA was used. mRNA purification was performed using the PolyATtract mRNA Isolation System III (Fisher) according to the manufacture's instruction.

### Genomic DNA preparations

Strains were grown in 50 ml YPD medium at 30°C overnight with shaking. The cells were washed three times with distilled water and harvested by centrifugation at 4000×g for 8 minutes. The cell pellet was frozen immediately at −80°C, lyophilized overnight, and stored at −20°C until genomic DNA was prepared using the CTAB protocol as described previously [110].

### Gene disruption and complementation

To disrupt the *MAT2* or *ZNF2* gene, an overlap PCR product was generated with the NAT or NEO marker amplified from plasmid pAI1 or pJAF1 [32], [68] and 5′ and 3′ flanking sequences of the *MAT2* or the *ZNF2* locus from strain JEC21α (967 bp and 859 bp, respectively). The PCR product was directly introduced into strains JEC21α, XL280α, JEC20**a**, or XL254**a** by biolistic transformation [111]. Mutants in which the gene had been replaced by homologous recombination were screened by PCR and confirmed by Southern blotting. The same approach with the NEO marker was used to produce deletion mutants in the serotype A H99 background with primers designed based on the H99 genome sequence. For complementation, an overlap PCR product with the NEO or NAT marker and the wild-type *ZNF2* gene containing its native promoter and terminator from strain JEC21 or H99 was generated. The PCR product was directly introduced into *znf2Δ* mutants in various backgrounds. Isogenic *MAT* **a** strains with the *ZNF2* deletion were obtained by selecting resistant *MAT* **a** progeny from a cross between the mutant α strains and the corresponding congenic pair strains (JEC20**a** or KN99**a**).

### Mating and self-filamentation assays

For matings, mating partners (**a** and α) isolates were grown on YPD medium separately overnight at 30°C and then cocultured together on V8 medium (pH = 7.0 for serotype D strains, pH = 5.0 for serotype A strains) in the dark at 22°C. Each partner was also grown alone on the same V8 medium plate as a control. Matings were examined microscopically for formation of mating hyphae and chains of basidiospores. Random basidiospores were isolated using a micromanipulator. Their mating type was examined by mating with reference strains JEC20 (**a**) or JEC21 (α). For self-filamentation assays (only for serotype D strains), cells were patched on V8 medium alone and hyphae formation was examined microscopically.

### Cell fusion assay

Strains were grown on YPD agar for 2 days, resuspended in sterile water, quantitated in a spectrophotometer, and diluted to 10^7^ cells/ml. Equal numbers of mating partners were mixed and 10 microliters of the mixture were dropped on V8 juice agar medium (pH = 7.0). After 15 hours of incubation in the dark at room temperature, cells were removed, washed, and plated on media to select fusion products at room temperature. CFU were counted to measure the efficiency of cell fusion. The *znf2Δ* mutant strains XL874**a** (*znf2*::*NAT^r^ lys1*) and XL875α (*znf2*::*NAT^r^ ade2*) were used for fusion assay and the corresponding control pairs were XL878**a** (*lys1*) and XL877α (*ade2*). Fused prototrophic products were selected on minimum YNB medium. The colony morphology of the fusion product from wild type and the *znf2Δ* mutant was also observed microscopically.

The *mat2Δ* mutants used for fusion assay were XL926α (*mat2*::*NAT^r^*) and XL961**a** (*mat2*::*NEO^r^*). The corresponding control pairs were JEC21α marked with NAT^r^ and JEC20**a** marked with NEO^r^. Fused products were selected on YPD+NAT+NEO medium. To assay cell fusion during unilateral matings, XL926α (*mat2*::*NAT^r^*) was paired with JEC20**a** marked with NEO^r^, and XL961**a** (*mat2*::*NEO^r^*) was paired with JEC21α marked with NAT^r^. Again, the fusion products were selected on YPD+NAT+NEO medium.

### Confrontation assay

Strains (**a** and α) were streaked in close proximity but not touching each other on Filamentation agar or V8 medium and incubated in the dark at 22°C. Formation of conjugation tubes or monokaryotic hyphae was examined after 24 hours.

### Ploidy determination by fluorescence flow cytometry

Cells were processed for flow cytometry as described previously [81]. Briefly, cells were harvested after overnight growth in YPD medium, washed once in PBS buffer, and fixed in 1 ml of 70% ethanol overnight at 4°C. Fixed cells were washed once with 1 ml of NS buffer (10 mM Tris–HCl (pH = 7.6), 250 mM sucrose, 1 mM EDTA (pH = 8.0), 1 mM MgCl_2_, 0.1 mM CaCl_2_, 0.1 mM ZnCl_2_) and then stained with propidium iodide (10 mg/ml) in 200 µl of NS buffer containing RNaseA (1 mg/ml) at 4°C for 4–16 h. Then 50 µl of stained cells was diluted into 2 ml of 50 mM Tris–HCl (pH = 8.0) and sonicated for 1 min. Flow cytometry was performed on 10,000 cells and analyzed on the FL1 channel with a Becton–Dickinson FACScan.

### *In vitro* assay of virulence factors

Virulence traits were assayed as previously described [81]. Briefly, yeast cells were grown in YPD medium overnight and washed three times with water. Cell density was determined by optical density at 600 nm and cells were serially diluted (×10). To analyze growth at different temperatures, cells were spotted on YPD medium and incubated at the indicated temperatures for 48 hours. To examine melanin production, cells were spotted on melanin-inducing media containing L-DOPA (L-dihydroxyphenylalanine, 100 mg/L) [112] and incubated at 22°C in the dark for 6 days. Melanization was observed as the colony developed a brown color. To characterize capsule production, equal numbers of *C. neoformans* cells were transferred to Dulbecco's Modified Eagle Medium (DMEM) (Invitrogen, California) and grown for three days at 37°C. Cells were then suspended in India ink. The capsule excludes ink particles and was visualized as a white halo surrounding the yeast cell.

### Murine inhalation model of cryptococcosis

Animals were infected essentially as previously described [113]. For strains in the serotype A H99 background, groups of 6- to 8-week-old female A/J mice were anesthetized by intraperitoneal injection of Phenobarbital (∼0.035 mg/g) and they were infected intranasally with 5×10^4^ *Cryptococcus* cells in 50 µl PBS. The inocula of yeast cells were confirmed by CFU after serial dilutions. After inoculation of fungal cells, animals were monitored twice daily, and those showing signs of severe morbidity (weight loss, extension of the cerebral portion of the cranium, abnormal gait, paralysis, seizures, convulsions, or coma) were sacrificed by CO_2_ inhalation. The survival rates of animals were plotted against time, and *P* values were calculated with the Mann-Whitney U test. For strains in various serotype D backgrounds, DBA mice were used and the inoculum was increased to 1×10^6^ *Cryptococcus* cells per animal. Other procedures were the same as described above.

## Supporting Information

Figure S1 Deletion of *STE12* reduces but does not abolish α-**a** mating. The indicated strains were co-incubated on V8 medium (pH 7.0) in the dark at 22°C for 48 hours. Deletion of *STE12* reduces α-**a** unilateral mating in both the wild type background and in the *znf2*Δ mutant background.
(0.44 MB TIF)

Figure S2 *ZNF2* is highly expressed in the hyperfilamentous strain XL280. The expression pattern of the *ZNF2* gene during self-filamentation in strain XL280 and XL34 that were cultured on V8 medium (pH = 7.0) for 24 hr.
(0.09 MB TIF)

Figure S3 Independent animal study with a modestly higher inoculation and fewer animals indicates that Mat2 and Ste7 behave similarly, while Znf2 is a negative regulator of pathogenicity. Animals (five to eight each group) were intranasally infected with 1×10^5^ yeast cells of the wild type (H99), *ste7*Δ (YSB345), *mat2*Δ (XL1598), *znf2*Δ (XL1601), and *znf2*Δ-*ZNF2* (XL1643) strains. Survival was plotted against time after inoculation. *P* values compared to the wild type control are: *ste7*Δ (*P* = 0.00085), *mat2*Δ (*P* = 0.00026), *znf2*Δ (*P*<0.0001 ), *znf2*Δ+*ZNF2* (*P* = <0.0001). The *P* value of *mat2*Δ group compared to *ste7*Δ group is 0.07153.
(0.33 MB TIF)

Figure S4 Classical virulence traits are not altered by *mat2* or *znf2* mutations. Yeast cells of *C. neoformans* strains (H99, XL1598, XL1601, XL1643, JEC21, XL576, XL910, XL280, XL574, XL904, XL254**a**, XL575**a** , and XL900**a**) were quantified by determining the optical density at 600 nm. Three-microliter serial dilutions (10-fold) of cells were spotted onto media for phenotypic characterization. (A) Cells were grown on YPD medium at 22°C for 3 days as a control for growth (first column from the left); cells were grown on YPD medium at 37°C for 3 days (second column); cells were grown on medium containing L-DOPA at 22°C for 6 days or 2 days for strains in the H99 background (third column); cells were grown on DME medium at 37°C for 3 days and become more mucoid when capsule is produced (fourth column). Capsule production was confirmed with India ink staining (data not shown).
(6.60 MB TIF)

Figure S5 Deletion of *STE12* reduces but does not abolish monokaryotic fruiting in the hyperfilamentous strain XL280. The indicated strains were incubated on V8 medium (pH 7.0) in the dark at 22°C for 7 days.
(0.18 MB TIF)

Table S1 Genes differentially expressed during bilateral **a**-α matings in *ste7*Δ and *mat2*Δ mutants in the JEC20**a**/JEC21α background.
(0.02 MB XLS)

Table S2 Genes differentially expressed during bilateral **a**-α matings in *znf2*Δ mutants in the JEC20**a**/JEC21α background.
(1.89 MB XLS)

Table S3 Genes differentially expressed during monokaryotic fruiting in *ste7*Δ, *mat2*Δ, and *znf2*Δ mutants in the XL280α background.
(0.04 MB XLS)

Table S4 List of strains used in this study.
(0.12 MB DOC)
