# Supplementary figures and images for: Transcription Factors Mat2 and Znf2 Operate Cellular Circuits Orchestrating Opposite- and Same-Sex Mating in Cryptococcus neoformans
Source: PLoS Genet. 2010 May 13;6(5):e1000953. doi: 10.1371/journal.pgen.1000953 (PMC2869318; doi:10.1371/journal.pgen.1000953)

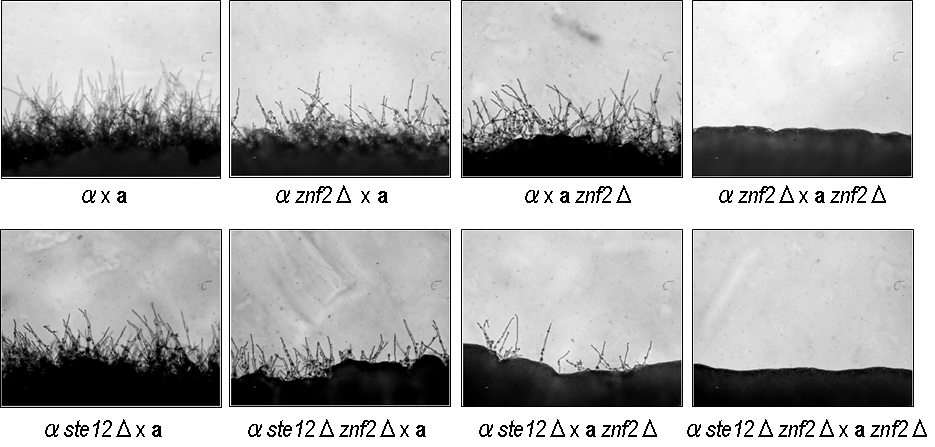

Supplement: Figure S1 — Deletion of STE12 reduces but does not abolish α-a mating. The indicated strains were co-incubated on V8 medium (pH 7.0) in the dark at 22°C for 48 hours. Deletion of STE12 reduces α-a unilateral mating in both the wild type background and in the znf2Δ mutant background. (0.44 MB TIF) [file pgen.1000953.s001.tif]

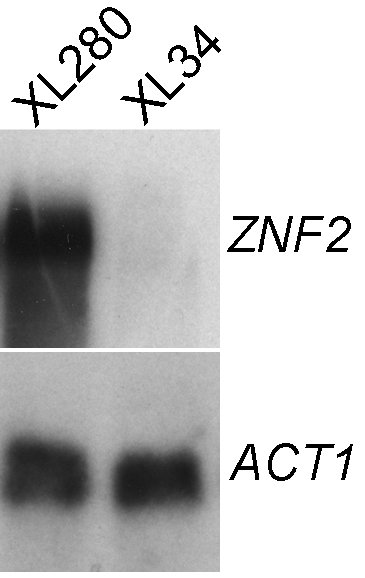

Supplement: Figure S2 — ZNF2 is highly expressed in the hyperfilamentous strain XL280. The expression pattern of the ZNF2 gene during self-filamentation in strain XL280 and XL34 that were cultured on V8 medium (pH = 7.0) for 24 hr. (0.09 MB TIF) [file pgen.1000953.s002.tif]

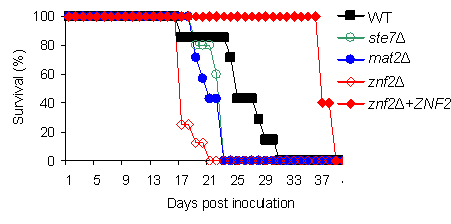

Supplement: Figure S3 — Independent animal study with a modestly higher inoculation and fewer animals indicates that Mat2 and Ste7 behave similarly, while Znf2 is a negative regulator of pathogenicity. Animals (five to eight each group) were intranasally infected with 1×10^5^ yeast cells of the wild type (H99), ste7Δ (YSB345), mat2Δ (XL1598), znf2Δ (XL1601), and znf2Δ-ZNF2 (XL1643) strains. Survival was plotted against time after inoculation. P values compared to the wild type control are: ste7Δ (P = 0.00085), mat2Δ (P = 0.00026), znf2Δ (P<0.0001 ), znf2Δ+ZNF2 (P = <0.0001). The P value of mat2Δ group compared to ste7Δ group is 0.07153. (0.33 MB TIF) [file pgen.1000953.s003.tif]

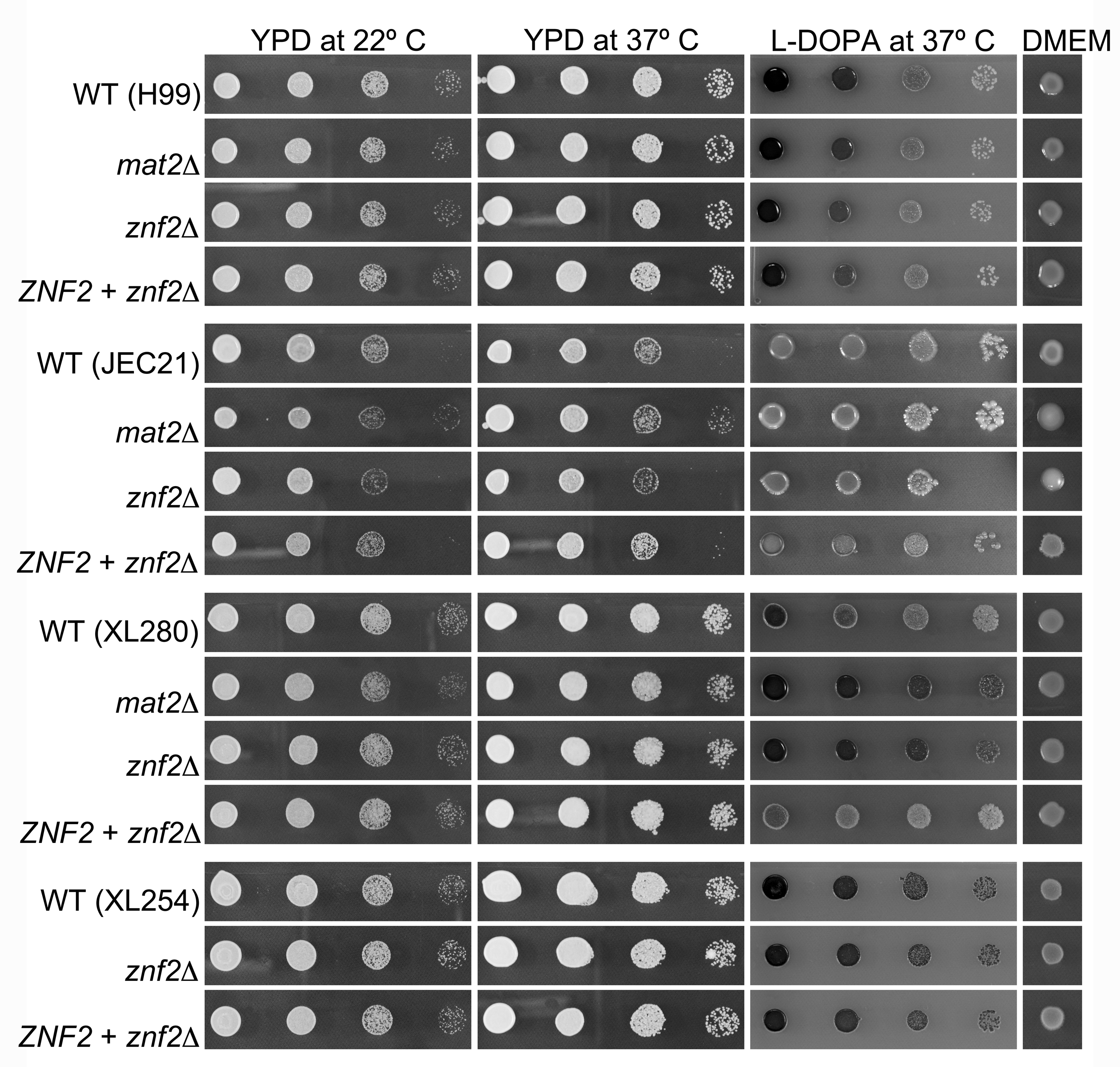

Supplement: Figure S4 — Classical virulence traits are not altered by mat2 or znf2 mutations. Yeast cells of C. neoformans strains (H99, XL1598, XL1601, XL1643, JEC21, XL576, XL910, XL280, XL574, XL904, XL254a, XL575a , and XL900a) were quantified by determining the optical density at 600 nm. Three-microliter serial dilutions (10-fold) of cells were spotted onto media for phenotypic characterization. (A) Cells were grown on YPD medium at 22°C for 3 days as a control for growth (first column from the left); cells were grown on YPD medium at 37°C for 3 days (second column); cells were grown on medium containing L-DOPA at 22°C for 6 days or 2 days for strains in the H99 background (third column); cells were grown on DME medium at 37°C for 3 days and become more mucoid when capsule is produced (fourth column). Capsule production was confirmed with India ink staining (data not shown). (6.60 MB TIF) [file pgen.1000953.s004.tif]

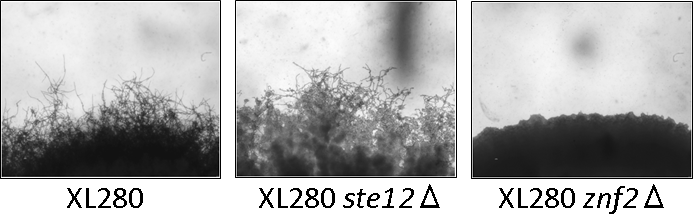

Supplement: Figure S5 — Deletion of STE12 reduces but does not abolish monokaryotic fruiting in the hyperfilamentous strain XL280. The indicated strains were incubated on V8 medium (pH 7.0) in the dark at 22°C for 7 days. (0.18 MB TIF) [file pgen.1000953.s005.tif]
