# Supplementary material for: Transcription Factors Mat2 and Znf2 Operate Cellular Circuits Orchestrating Opposite- and Same-Sex Mating in Cryptococcus neoformans
Source: PLoS Genet. 2010 May 13;6(5):e1000953. doi: 10.1371/journal.pgen.1000953 (PMC2869318; doi:10.1371/journal.pgen.1000953)
Supplement: Table S4 — List of strains used in this study. (0.12 MB DOC) [file pgen.1000953.s009.doc]

**Table S4. Strains used in this study**

| **Strain name** | **Genotype** | **Source and comments** |
| --- | --- | --- |
| JEC21α | wild type | [116,117] |
| JEC20**a** | wild type | Congenic with JEC21 [116,117] |
| JEC169**a** ^1^ | *ade2 lys1 ura5* | [118] |
| RDC7**a** ^1^ | *ade2 cpk1::ADE2* | [4] |
| XL576α ^1^ | *znf2*::*NAT^r^* | this study |
| XL578α ^1^ | *znf2*::*NAT^r^* | this study |
| XL867**a** ^1^ | *znf2*::*NAT^r^* | From a cross between JEC169**a** and XL578 |
| XL872α ^1^ | *znf2*::*NAT^r^ ade2 lys1* | from a cross between JEC169**a** and XL576α |
| XL873α ^1^ | *znf2*::*NAT^r^ ura5* | from a cross between JEC169**a** and XL576α |
| XL877α ^1^ | *ade2* | from a cross between JEC169**a** and XL576α |
| XL878**a** ^1^ | *lys1* | from a cross between JEC169**a** and XL576α |
| XL874**a** ^1^ | *znf2*::*NAT^r^* *lys1* | this study |
| XL875α ^1^ | *znf2*::*NAT^r^* *ade2* | this study |
| XL879**a** ^1^ | *znf2*::*NAT^r^* | this study |
| XL910α ^1^ | *znf2*::*NAT^r^* *ZNF2-NEO^r^* | complementation of XL576 |
| XL978α ^1^ | *znf2*::*NAT^r^ ura5* | 5-FOA^r^ isolate of XL576 |
| XL1059**a** ^1^ | *znf2*::*NAT^r^ ura5* | 5-FOA^r^ isolate of XL879 |
| XL1134 α/α ^1^ | *znf2*::*NAT^r^ znf2*::*NAT^r^* | fusion product between XL872 with XL873 in the presence of JEC169. Diploid by FACS. |
| XL1131α ^1^ | *ade2 cpk1::ADE2 znf2::NAT^r^* | from a cross between RDC7 and XL576 |
| XL1127**a** ^1^ | *znf2::NAT^r^ ura5 URA5 P_GPD1_-SXI1α* | XL1059 transformed with pCH258 [39,40] that bears the *SXI1α* gene with the *GPD1* promoter. |
| XL926α ^1^ | *mat2::NAT^r^* | this study |
| XL961**a** ^1^ | *mat2::NEO^r^* | this study |
| XL975α ^1^ | *mat2::NAT^r^ ura5* | 5-FOA^r^ isolate of XL926 |
| XL1073 **a** ^1^ | *mat2::NEO^r^ ura5* | 5-FOA^r^ isolate of XL961 |
| XL1101 **a** ^1^ | *mat2::NAT^r^ ura5 URA5 P_GPD1_-SXI1* | XL1073 transformed with pCH258 [39,40] that bears the *SXI1α* gene with the *GPD1* promoter. |
| XL948α ^1^ | *ste7::NAT^r^* | this study |
| XL951**a** ^1^ | *ste7::NAT^r^* | this study |
| XL984α ^1^ | *ste7::NAT^r^ ura5* | 5-FOA^r^ isolate of XL948 |
| XL1062**a** ^1^ | *ste7::NATr ura5* | 5-FOA^r^ isolate of XL951 |
| XL1118**a** ^1^ | *ste7::**NAT^r^ ura5 URA5 P_GPD1_-SXI1α* | XL1062 transformed with pCH258 [39,40] that bears the *SXI1* gene with the *GPD1* promoter. |
| CFW5α ^1^ | *ste12α:: NAT^r^* | this study |
| MF5α ^1^ | *ste12α:: NAT^r^ znf2:: NAT^r^* | from a cross between CFW5 and XL867 |
| H99 | wild type | [119] |
| KN99α ^2^ | wild type | Isogenic with H99 [31] |
| KN99**a** ^2^ | wild type | Congenic with H99/KN99α [31] |
| YSB119α ^2^ | *aca1*::*NAT^r^* *ura5* *ACA1-URA5* | [120] |
| YSB121**a** ^2^ | *aca1*::*NEO^r^* *ura5* *ACA1-URA5* | [120] |
| YSB345α ^2^ | *ste7::NEO^r^* | From Y. S. Bahn |
| XL1598α ^2^ | *mat2::NEO^r^* |  |
| XL1601α ^2^ | *znf2*::*NEO^r^* | this study |
| XL1643α ^2^ | *znf2*::*NEO^r^* *ZNF2-NAT^r^* | complementation of XL1601 |
| XL280α | wild type | [35] |
| XL574α ^3^ | *znf2*::*NAT^r^* | this study |
| XL904α ^3^ | *znf2*::*NAT^r^* *ZNF2-NEO^r^* | complementation of XL574 |
| XL942α ^3^ | *mat2::NAT^r^* | this study |
| XL967α ^3^ | *mat2::NAT^r^ ura5* | 5-FOA^r^ isolate of XL942 |
| XL946α ^3^ | *ste7::NAT^r^* | this study |
| XL993α ^3^ | *ste7::NAT^r^ ura5* | 5-FOA^r^ isolate of XL946 |
| CFW4α ^3^ | *ste12*α::*NAT^r^* | this study |
| XL254**a** | wild type | [35] |
| XL575**a** ^4^ | *znf2*::*NAT^r^* | this study |
| XL900**a** ^4^ | *znf2*::*NAT^r^* *ZNF2-NEO^r^* | complementation of XL575 |
| XL987**a** ^4^ | *znf2*::*NAT^r^ ura5* | 5-FOA^r^ isolate of XL575 |
| XL187**a** | wild type | [35] |
| XL1445**a** ^5^ | *znf2*::*NAT^r^* | this study |
| XL304α | wild type | [35] |
| XL1447α ^6^ | *znf2*::*NAT^r^* | this study |

^1^: Strains in JEC21/JEC20 background (serotype D)

^2^: Strains in H99 background (serotype A)

^3^: Strains in XL280 background (serotype D)

^4^: Strains in XL254 background (serotype D)

^5^: Strains in XL187 background (serotype D)

^6^: Strains in XL304 background (serotype D)
